# Supplementary material for: Prognostic model of in-hospital ischemic stroke mortality based on an electronic health record cohort in Indonesia
Source: PLoS One. 2024 Jun 12;19(6):e0305100. doi: 10.1371/journal.pone.0305100 (PMC11168658; doi:10.1371/journal.pone.0305100)
Supplement: S1 File — (PDF) [file pone.0305100.s001.pdf]

Supplementary Materials

Post-Stroke Mortality in the National Brain Centre Hospital

Variable Selection

Feature Engineering

We started with a dataset of 3561 rows and 78 columns. The dataset contains following variables:

```r
names(tbl)
```

```
 [1] "sex_ps"            "umur_ps"           "tgl_admisi"
 [4] "jam_admisi"        "st_nikah"          "etnis"
 [7] "pekerjaan"         "pendidikan"        "alamat"
[10] "ALAMAO"            "kelurahan"         "kecamatan"
[13] "kota"              "diagnosa_sek"      "DIAGNO"
[16] "onset"             "tindakan"          "dtn"
[19] "riw_stroke_tia"    "thn_riw_stroke"    "jenis_riw_stroke"
[22] "riw_ht"            "riw_dm"            "obt_rutin"
[25] "riw_jantung"       "riw_ginjal"        "merokok"
[28] "alkohol"           "stroke_klg"        "E"
[31] "M"                 "V"                 "sistol"
[34] "diastol"           "GDS"               "komplikasi_rawat"
[37] "d_dimer"           "trigliserida"      "hdl"
[40] "ldl"               "kol_total"         "as_urat"
[43] "GDP"               "G2PP"              "HBA1C"
[46] "Hb"                "Ht"                "Leukosit"
[49] "Trombosit"         "nihss_msk"         "mrs_keluar"
[52] "imt"               "ekg"               "lama_rawat"
[55] "outcome"           "ct_scan"           "CT_SCO"
[58] "foto_thorax"       "FOTO_O"            "mri_brain"
```

```
[61] "MRI_BO"              "transformasi"        "stroke_in_evolution"
[64] "kelas_rawat"         "pembayaran"          "kelas_bpjs"
[67] "covid"               "riw_sakit_lainnya"   "RIW_SO"
[70] "keterangan"          "death"               "DM"
[73] "DM.uncontrolled"     "heart.disease"       "HT"
[76] "HT.uncontrolled"     "renal.disease"       "V.coherent"
```

In order to select suitable parameters to model the survivability of stroke patients, we initiated by analyzing the structure of our dataset. The complete dataset is a bit messy, with various mistyped information. For instance, there is a recorded value of a systolic blood pressure of 1160 mmHg, presumably a mistyped of 160 mmHg. As to not impose any further assumptions, we deleted entries which seems to be physiologically impossible. In the end, we included the following variables in our exploratory analysis:

- `outcome`: Outcome (0: Discharged, 1: Deceased)
- `umur_ps`: Patient's age in year
- `sex_ps`: Patient's sex (1: Female, 2: Male)
- `heart.disease`: Heart disease (0: False, 1: True)
- `renal.disease`: Renal disease
- `DM.uncontrolled`: Uncontrolled diabetes mellitus
- `HT.uncontrolled`: Uncontrolled hypertension
- `sistol`: Systolic blood pressure
- `diastol`: Diastolic blood pressure
- `E`: Eye (from GCS)
- `M`: Movement (from GCS)
- `V.coherent`: Coherent verbal speech
- `as_urat`: Uric acid level
- `imt`: BMI (body mass index)
- `lama_rawat`: Length of stay
- `nihss_msk`: NIHSS on admission

Upon selecting the variable of interest, we proceeded by evaluating the `null` (missing) entry.

```
feat.name <- c( # Feature of interest
  "outcome", "umur_ps", "sex_ps", "heart.disease", "renal.disease",
  "DM.uncontrolled", "HT.uncontrolled", "sistol", "diastol",
  "E", "M", "V.coherent", "as_urat", "imt", "lama_rawat", "nihss_msk"
)

t.lo <- list("systole" = 70, "diastole" = 30) # Clean systole and diastole cols

sub_tbl <- tbl %>% # Subsetting feature of interest
```

```r
  subset(.$sistol > t.lo$systole & .$diastol > t.lo$diastol, select=feat.name) %>%
  lapply(as.numeric) %>%
  data.frame()

missing <- sapply(sub_tbl, \(varname) is.na(varname) %>% sum()) %T>% print()
```

```
        outcome          umur_ps           sex_ps    heart.disease    renal.disease 
              0                0                0                0                0 
DM.uncontrolled HT.uncontrolled           sistol          diastol                E 
              2                0                0                0                0 
              M       V.coherent          as_urat              imt       lama_rawat 
              0                0              217               80                0 
      nihss_msk 
              0 
```

```r
round(missing / nrow(tbl), 2)
```

```
        outcome          umur_ps           sex_ps    heart.disease    renal.disease 
           0.00             0.00             0.00             0.00             0.00 
DM.uncontrolled HT.uncontrolled           sistol          diastol                E 
           0.00             0.00             0.00             0.00             0.00 
              M       V.coherent          as_urat              imt       lama_rawat 
           0.00             0.00             0.06             0.02             0.00 
      nihss_msk 
           0.00 
```

It is notable that `as_urat` variable had the most missing values (217; at 6%), while `imt` was the second most (80; at 2%). Cross tabulating the missing values with outcome status resulted in the following matrices. Please note that the second cross-tables report the percentage of missing value grouped by the outcome.

```r
missing_var <- missing %>%
  {.[. > 0]} %>%
  names() %>%
  set_names(., .)

missing_outcome <- missing_var %>%
  lapply(function(varname) {
    form <- sprintf("outcome ~ is.na(%s)", varname) %>% as.formula()
    ftable(form, data = sub_tbl)
```

```r
  }) %T>%
  print()
```

```
$DM.uncontrolled
                      outcome    1    2
is.na(DM.uncontrolled)
                  FALSE       3380  166
                  TRUE           2    0

$as_urat
              outcome    1    2
is.na(as_urat)
          FALSE       3211  120
          TRUE         171   46

$imt
          outcome    1    2
is.na(imt)
      FALSE       3315  153
      TRUE          67   13
```

```r
missing_outcome %>%
  lapply(\(mtx) round(mtx / rowSums(mtx), 2))
```

```
$DM.uncontrolled
                      outcome    1    2
is.na(DM.uncontrolled)
                  FALSE       0.95 0.05
                  TRUE        1.00 0.00

$as_urat
              outcome    1    2
is.na(as_urat)
          FALSE       0.96 0.04
          TRUE        0.79 0.21

$imt
          outcome    1    2
is.na(imt)
      FALSE       0.96 0.04
      TRUE        0.84 0.16
```

```r
lapply(missing_outcome, fisher.test)
```

```
$DM.uncontrolled

    Fisher's Exact Test for Count Data

data:  X[[i]]
p-value = 1
alternative hypothesis: true odds ratio is not equal to 1
95 percent confidence interval:
   0.0000 108.8572
sample estimates:
odds ratio
         0


$as_urat

    Fisher's Exact Test for Count Data

data:  X[[i]]
p-value < 2.2e-16
alternative hypothesis: true odds ratio is not equal to 1
95 percent confidence interval:
  4.834518 10.569604
sample estimates:
odds ratio
   7.18869


$imt

    Fisher's Exact Test for Count Data

data:  X[[i]]
p-value = 6.546e-05
alternative hypothesis: true odds ratio is not equal to 1
95 percent confidence interval:
 2.080726 7.889339
sample estimates:
odds ratio
  4.200846
```

These findings imply that patients with missing values on `as_urat` (uric acid level) or `imt` (BMI) had a greater odds of death. This, of course, does not imply any clinical relevance on such patients. However, from an analytic perspective, we can expect a bias when we remove these null entries.

```r
cat_vars <- subset(sub_tbl, select = c(3:7, 10:12)) %>% names()

explore_null <- function(missing_var, feat_name, fn, merge = FALSE, ...) {
  missing_var %>% set_names(., .) %>%
    lapply(function(varname) {
      exploration <- feat_name %>% set_names(., .) %>% lapply(function(feat) {
        if (varname != feat) {
          form <- sprintf("%s ~ is.na(%s)", feat, varname) %>% as.formula()
          fn(form, ...)
        }
      }) %>%
        {.[!sapply(., is.null)]}
      if (merge) {
        exploration %<>%
          reshape::merge_recurse(by = sprintf("is.na(%s)", varname))
      }
      return(exploration)
    })
}

explore_null(missing_var, cat_vars, ftable, data = sub_tbl) %T>%
  print() %>%
  lapply(function(varname) {
    lapply(varname, \(feat) round(feat / rowSums(feat), 2))
  })
```

```
$DM.uncontrolled
$DM.uncontrolled$sex_ps
                       sex_ps    1    2
is.na(DM.uncontrolled)
FALSE                         1287 2259
TRUE                             2    0

$DM.uncontrolled$heart.disease
                       heart.disease    0    1
is.na(DM.uncontrolled)
FALSE                                2532 1014
```

```
TRUE                                   1    1

$DM.uncontrolled$renal.disease
                       renal.disease    0    1
is.na(DM.uncontrolled)
FALSE                               3112  434
TRUE                                   2    0

$DM.uncontrolled$HT.uncontrolled
                       HT.uncontrolled    0    1
is.na(DM.uncontrolled)
FALSE                                 1145 2401
TRUE                                     1    1

$DM.uncontrolled$E
                       E    0    1    2    3    4    5   34
is.na(DM.uncontrolled)
FALSE                       1   22   61  204 3256    1    1
TRUE                        0    0    0    0    2    0    0

$DM.uncontrolled$M
                       M    0    1    2    3    4    5    6
is.na(DM.uncontrolled)
FALSE                       1    9    4    6   50  292 3184
TRUE                        0    0    0    0    0    0    2

$DM.uncontrolled$V.coherent
                       V.coherent    0    1
is.na(DM.uncontrolled)
FALSE                              525 3021
TRUE                                 0    2


$as_urat
$as_urat$sex_ps
               sex_ps    1    2
is.na(as_urat)
FALSE                 1204 2127
TRUE                    85  132

$as_urat$heart.disease
               heart.disease    0    1
is.na(as_urat)
```

```
FALSE                       2403  928
TRUE                         130   87

$as_urat$renal.disease
               renal.disease    0    1
is.na(as_urat)
FALSE                       2946  385
TRUE                         168   49

$as_urat$DM.uncontrolled
               DM.uncontrolled    0    1
is.na(as_urat)
FALSE                         2230 1099
TRUE                           146   71

$as_urat$HT.uncontrolled
               HT.uncontrolled    0    1
is.na(as_urat)
FALSE                         1042 2289
TRUE                           104  113

$as_urat$E
               E    0    1    2    3    4    5   34
is.na(as_urat)
FALSE               1   14   45  185 3085    1    0
TRUE                0    8   16   19  173    0    1

$as_urat$M
               M    0    1    2    3    4    5    6
is.na(as_urat)
FALSE               1    6    4    5   35  265 3015
TRUE                0    3    0    1   15   27  171

$as_urat$V.coherent
               V.coherent    0    1
is.na(as_urat)
FALSE                      470 2861
TRUE                        55  162


$imt
$imt$sex_ps
           sex_ps    1    2
```

```
is.na(imt)
FALSE            1258 2210
TRUE               31   49

$imt$heart.disease
          heart.disease    0    1
is.na(imt)
FALSE                   2471  997
TRUE                      62   18

$imt$renal.disease
          renal.disease    0    1
is.na(imt)
FALSE                   3048  420
TRUE                      66   14

$imt$DM.uncontrolled
          DM.uncontrolled    0    1
is.na(imt)
FALSE                     2319 1147
TRUE                        57   23

$imt$HT.uncontrolled
          HT.uncontrolled    0    1
is.na(imt)
FALSE                     1112 2356
TRUE                        34   46

$imt$E
          E    0    1    2    3    4    5   34
is.na(imt)
FALSE          1   20   58  198 3190    1    0
TRUE           0    2    3    6   68    0    1

$imt$M
          M    0    1    2    3    4    5    6
is.na(imt)
FALSE          1    7    4    6   48  282 3120
TRUE           0    2    0    0    2   10   66

$imt$V.coherent
          V.coherent    0    1
is.na(imt)
```

```
FALSE                  508 2960
TRUE                    17   63


$DM.uncontrolled
$DM.uncontrolled$sex_ps
                       sex_ps    1    2
is.na(DM.uncontrolled)
FALSE                         0.36 0.64
TRUE                          1.00 0.00

$DM.uncontrolled$heart.disease
                       heart.disease    0    1
is.na(DM.uncontrolled)
FALSE                                0.71 0.29
TRUE                                 0.50 0.50

$DM.uncontrolled$renal.disease
                       renal.disease    0    1
is.na(DM.uncontrolled)
FALSE                                0.88 0.12
TRUE                                 1.00 0.00

$DM.uncontrolled$HT.uncontrolled
                       HT.uncontrolled    0    1
is.na(DM.uncontrolled)
FALSE                                  0.32 0.68
TRUE                                   0.50 0.50

$DM.uncontrolled$E
                       E    0    1    2    3    4    5   34
is.na(DM.uncontrolled)
FALSE                    0.00 0.01 0.02 0.06 0.92 0.00 0.00
TRUE                     0.00 0.00 0.00 0.00 1.00 0.00 0.00

$DM.uncontrolled$M
                       M    0    1    2    3    4    5    6
is.na(DM.uncontrolled)
FALSE                    0.00 0.00 0.00 0.00 0.01 0.08 0.90
TRUE                     0.00 0.00 0.00 0.00 0.00 0.00 1.00

$DM.uncontrolled$V.coherent
                       V.coherent    0    1
```

```
is.na(DM.uncontrolled)
FALSE                          0.15 0.85
TRUE                           0.00 1.00


$as_urat
$as_urat$sex_ps
              sex_ps    1    2
is.na(as_urat)
FALSE               0.36 0.64
TRUE                0.39 0.61

$as_urat$heart.disease
              heart.disease    0    1
is.na(as_urat)
FALSE                      0.72 0.28
TRUE                       0.60 0.40

$as_urat$renal.disease
              renal.disease    0    1
is.na(as_urat)
FALSE                      0.88 0.12
TRUE                       0.77 0.23

$as_urat$DM.uncontrolled
              DM.uncontrolled    0    1
is.na(as_urat)
FALSE                        0.67 0.33
TRUE                         0.67 0.33

$as_urat$HT.uncontrolled
              HT.uncontrolled    0    1
is.na(as_urat)
FALSE                        0.31 0.69
TRUE                         0.48 0.52

$as_urat$E
              E    0    1    2    3    4    5   34
is.na(as_urat)
FALSE           0.00 0.00 0.01 0.06 0.93 0.00 0.00
TRUE            0.00 0.04 0.07 0.09 0.80 0.00 0.00

$as_urat$M
```

```
               M    0    1    2    3    4    5    6
is.na(as_urat)
FALSE            0.00 0.00 0.00 0.00 0.01 0.08 0.91
TRUE             0.00 0.01 0.00 0.00 0.07 0.12 0.79

$as_urat$V.coherent
               V.coherent    0    1
is.na(as_urat)
FALSE                     0.14 0.86
TRUE                      0.25 0.75


$imt
$imt$sex_ps
           sex_ps    1    2
is.na(imt)
FALSE             0.36 0.64
TRUE              0.39 0.61

$imt$heart.disease
           heart.disease    0    1
is.na(imt)
FALSE                    0.71 0.29
TRUE                     0.78 0.22

$imt$renal.disease
           renal.disease    0    1
is.na(imt)
FALSE                    0.88 0.12
TRUE                     0.82 0.17

$imt$DM.uncontrolled
           DM.uncontrolled    0    1
is.na(imt)
FALSE                      0.67 0.33
TRUE                       0.71 0.29

$imt$HT.uncontrolled
           HT.uncontrolled    0    1
is.na(imt)
FALSE                      0.32 0.68
TRUE                       0.42 0.58
```

```
$imt$E
          E    0    1    2    3    4    5   34
is.na(imt)
FALSE       0.00 0.01 0.02 0.06 0.92 0.00 0.00
TRUE        0.00 0.03 0.04 0.07 0.85 0.00 0.01

$imt$M
          M    0    1    2    3    4    5    6
is.na(imt)
FALSE       0.00 0.00 0.00 0.00 0.01 0.08 0.90
TRUE        0.00 0.03 0.00 0.00 0.03 0.12 0.82

$imt$V.coherent
          V.coherent    0    1
is.na(imt)
FALSE                0.15 0.85
TRUE                 0.21 0.79
```

```
explore_null(missing_var, feat.name, merge = TRUE, aggregate, data = sub_tbl, median)
```

```
$DM.uncontrolled
  is.na(DM.uncontrolled) outcome umur_ps sex_ps heart.disease renal.disease
1                  FALSE       1    59.0      2           0.0             0
2                   TRUE       1    69.5      1           0.5             0
  HT.uncontrolled sistol diastol E M V.coherent as_urat  imt lama_rawat
1             1.0    155    90.0 4 6          1    5.70 24.6          4
2             0.5    180   101.5 4 6          1    7.55 28.5         10
  nihss_msk
1       6.0
2       4.5

$as_urat
  is.na(as_urat) outcome umur_ps sex_ps heart.disease renal.disease
1          FALSE       1      59      2             0             0
2           TRUE       1      60      2             0             0
  DM.uncontrolled HT.uncontrolled sistol diastol E M V.coherent  imt lama_rawat
1               0               1    157      90 4 6          1 24.6          4
2               0               1    140      81 4 6          1 24.0          5
  nihss_msk
1         6
2         8
```

```
$imt
  is.na(imt) outcome umur_ps sex_ps heart.disease renal.disease DM.uncontrolled
1      FALSE       1      59      2             0             0               0
2       TRUE       1      59      2             0             0               0
  HT.uncontrolled sistol diastol E M V.coherent as_urat lama_rawat nihss_msk
1               1    155      90 4 6          1     5.7          4         6
2               1    159      86 4 6          1     5.0          3         7
```

```
explore_null(missing_var, feat.name, merge = TRUE, aggregate, data = sub_tbl, mean) %>%
  lapply(round, 2)
```

```
$DM.uncontrolled
  is.na(DM.uncontrolled) outcome umur_ps sex_ps heart.disease renal.disease
1                      0    1.05   59.26   1.64          0.29          0.12
2                      1    1.00   69.50   1.00          0.50          0.00
  HT.uncontrolled sistol diastol   E    M V.coherent as_urat   imt lama_rawat
1            0.68 156.55   89.34 3.9 5.87       0.85    5.96 25.04       5.19
2            0.50 180.00  101.50 4.0 6.00       1.00    7.55 28.50      10.00
  nihss_msk
1      6.55
2      4.50

$as_urat
  is.na(as_urat) outcome umur_ps sex_ps heart.disease renal.disease
1              0    1.04   59.29   1.64          0.28          0.12
2              1    1.21   58.86   1.61          0.40          0.23
  DM.uncontrolled HT.uncontrolled sistol diastol    E    M V.coherent   imt
1            0.33            0.69 157.51   89.69 3.90 5.88       0.86 25.07
2            0.33            0.52 142.15   84.01 3.79 5.65       0.75 24.51
  lama_rawat nihss_msk
1       5.11      6.43
2       6.40      8.29

$imt
  is.na(imt) outcome umur_ps sex_ps heart.disease renal.disease DM.uncontrolled
1          0    1.04   59.30   1.64          0.29          0.12            0.33
2          1    1.16   57.76   1.61          0.22          0.17            0.29
  HT.uncontrolled sistol diastol    E    M V.coherent as_urat lama_rawat
1            0.68 156.64   89.38 3.89 5.87       0.85    5.97       5.19
2            0.58 153.51   87.71 4.15 5.70       0.79    5.56       5.24
```

```
  nihss_msk
1      6.52
2      7.82
```

Exploring the data above gave an indication that the missingness in `DM.uncontrolled`, `as_urat`, and `imt` may happen not at random.

```
clean_tbl <- sub_tbl %>% na.omit()
```

Afterwards, we calculate Mahalanobis distance, denoting individual distance from the distribution.

```
clean_tbl$outcome %<>% subtract(1) # 0 = discharged, 1 = death

feature <- clean_tbl %>% # Min-Max regularization for all feature of interest
  subset(select=-c(outcome, lama_rawat)) %>%
  lapply(function(feat) {
    as.numeric(feat) %>%
      {(. - min(., na.rm=TRUE)) / (max(., na.rm=TRUE) - min(., na.rm=TRUE))}
  }) %>%
  data.frame() %>%
  tibble::tibble()

feat_mean    <- colMeans(feature)
feat_cov     <- cov(feature, use = "pairwise.complete.obs")
clean_tbl$dist <- mahalanobis(feature, feat_mean, feat_cov)
```

Fitting Logistic Regression Models

```
varname <- names(feature) # Get variable names of interest

parsimonius <- c( # Parsimonious variables, obtained by heuristically fitting a model
  "outcome", "sex_ps", "umur_ps", "heart.disease", "renal.disease", "imt",
  "E", "M", "V.coherent"
)

dats <- list( # List containing all variable combinations for data mining process
  clean_tbl,
  subset(tbl,      select = c("outcome", varname)),
  subset(clean_tbl, select = c("outcome", varname)),
```

```r
  subset(tbl,     select = c("outcome", varname)),
  subset(tbl,     select = c("outcome", "nihss_msk")),
  subset(clean_tbl, select = c("outcome", varname, "dist")),
  subset(
    tbl, select = c(
      "outcome", varname[!varname %in% c("sistol", "diastol", "as_urat")]
    )
  )
) %>% set_names(paste0("dat", 1:length(.)))
```

When fitting a multivariable logistic regression model, we used stepwise method with a bidirectional approach, i.e. using both forward and backward algorithm.

```r
logregs <- lapply(dats, function(dat) { # Iterate all logreg models
  dat %<>% na.omit()
  minmod  <- glm(outcome ~ 1, data = dat, family = binomial)
  maxform <- glm(outcome ~ ., data = dat, family = binomial) %>% formula()
  step(minmod, scope = maxform, direction = "both")
})

rsq.mods <- c() # Find the R-square in best-fitting models

for (mod in logregs) {
  print(summary(mod))
  id <- length(rsq.mods) + 1
  rsq.mods[[id]] <- glm(mod$formula, data = mod$data, family = binomial) %>%
    rsq::rsq() %T>%
    message()
}

rsq.mods %<>% set_names(names(logregs))
rm(list = c("id", "mod"))

pars.tbl <- tbl %>% subset(select = parsimonius) %>% na.omit() %>%
  inset(c("E", "M"), value = {list(
    .$E %>% {ifelse(. == 0, 1, .)} %>% factor(levels = 5:1),
    .$M %>% {ifelse(. == 0, 1, .)} %>% factor(levels = 6:1)
  )})

par(mfrow = c(2, 2))
logreg <- glm(outcome ~ ., data = pars.tbl, family = binomial) %T>%
```

```
  {print(summary(.))} %T>%
  {print(rsq::rsq(.))} %T>%
  plot()
```

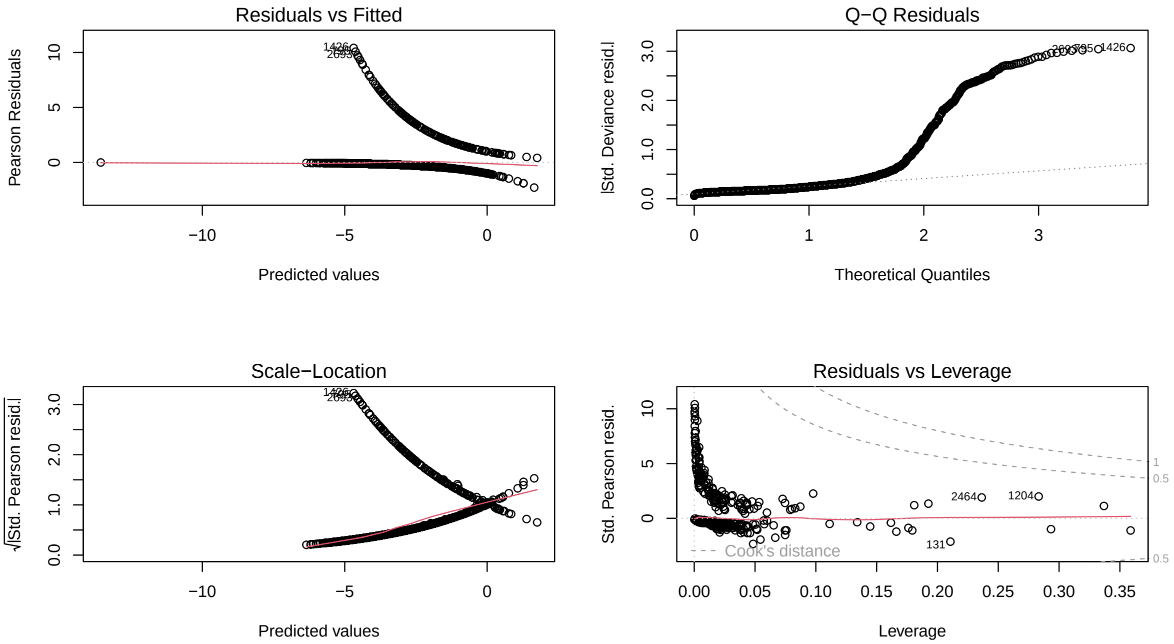


```
rsq.mods %<>% inset2("parsim", value = rsq::rsq(logreg)) %>% unlist()
```

Evaluating Logistic Regression Models

```
evalMod <- function(mod, ...) { # Evaluate a GLM binomial model
  outcome <- mod$data$outcome
  if (is.factor(outcome)) {outcome <- as.numeric(outcome) - 1}
  predicted <- {predict(mod, type = "response") > 0.5} %>% as.numeric()

  #Compare the actual and predicted value
  data.frame(
    "auc"       = ModelMetrics::auc(mod),                      # 1: best
    "precision" = ModelMetrics::precision(outcome, predicted), # 1: best
    "f1score"   = ModelMetrics::f1Score(outcome, predicted),   # 1: best
    "brier"     = ModelMetrics::brier(mod),                    # 0: best
```

```r
    "rmsle"     = ModelMetrics::rmsle(mod)                          # 0: best
  )
}
```

To evaluate all logistic regression models, we are using several metrics in deciding which model perform the best:

- Area under the curve
- Precision score
- F1 score
- Brier's score
- Random mean squared logarithmic error

```r
mod.evals <- rbind( # Model evaluation
  "ref"    = evalMod(glm(outcome ~ 1, data = clean_tbl, family = binomial)),
  "parsim" = evalMod(logreg),
  lapply(logregs, evalMod) %>% {do.call(rbind, .)}
) %>% inset2("bss", value = { # Calculate the brier skill score
  1 - {.$brier / .$brier[1]} # 1: best
}) %T>%
  print()
```

```
             auc precision    f1score      brier     rmsle         bss
ref    0.5000000 0.0000000 0.00000000 0.03356784 0.1273946  0.00000000
parsim 0.8629480 0.3888889 0.14736842 0.03652605 0.1332940 -0.08812614
dat1   0.8819837 0.3500000 0.10447761 0.02933418 0.1193396  0.12612244
dat2   0.8742321 0.3333333 0.09090909 0.02929263 0.1193178  0.12736045
dat3   0.8743928 0.3333333 0.09090909 0.02936051 0.1194553  0.12533829
dat4   0.8742321 0.3333333 0.09090909 0.02929263 0.1193178  0.12736045
dat5   0.7863727 0.5000000 0.06703911 0.04130548 0.1419334 -0.23050754
dat6   0.8749279 0.3478261 0.11678832 0.02917646 0.1190549  0.13082122
dat7   0.8709985 0.4444444 0.16842105 0.03596447 0.1320135 -0.07139650
```

```r
votes <- data.frame( # Vote for the best performing model
  "rsq" = rsq.mods %>% {names(.)[which(. == max(.))]},
  mapply(function(metrics, func) {
    to.parse <- parse(text = sprintf("metrics == %s(metrics)", func))
    metrics %>% {rownames(mod.evals)[which(eval(to.parse))]}
  },
    metrics  = mod.evals,
    func     = list("max", "max", "max", "min", "min", "max"),
```

```r
      SIMPLIFY = FALSE
    )
  ) %T>%
    print()
```

```
   rsq  auc precision f1score brier rmsle  bss
1 dat7 dat1      dat5    dat7  dat6  dat6 dat6
```

```r
winner <- votes %>% # Get the winning model
  unlist() %>%
  table() %T>%
  print() %>%
  {names(.)[which(. == max(.))]}
```

```
.
dat1 dat5 dat6 dat7
   1    1    3    2
```

```r
winpred <- logregs %>% # Get the winning predictors
  extract2(winner) %>%
  extract2("formula") %>%
  terms() %>%
  labels()

logregs %>% # Summary of the winning model
  extract2(winner) %>%
  gtsummary::tbl_regression(exponentiate = TRUE) %>%
  gtsummary::as_hux_table()
```

Table 1: Summary of the winning model

Characteristic	OR	95% CI	p-value
nihss_msk	1.13	1.08, 1.17	<0.001
heart.disease	2.52	1.61, 3.97	<0.001
E	0.47	0.31, 0.71	<0.001
renal.disease	2.42	1.50, 3.90	<0.001
imt	0.93	0.87, 0.99	0.023
umur_ps	1.03	1.01, 1.05	0.006
M	0.48	0.31, 0.72	<0.001
sex_ps	1.50	0.95, 2.40	0.085
dist	0.98	0.96, 0.99	0.010
as_urat	1.10	1.02, 1.17	0.008

OR = Odds Ratio, CI = Confidence Interval

Survival Analysis

Dataset Description

In the previous analysis, we used the subset of the raw data. In this subset, we have discarded any `null` values, resulting in the following data frame structure:

```
'data.frame':   3278 obs. of  17 variables:
 $ outcome        : num  0 0 0 0 0 0 0 0 0 0 ...
 $ umur_ps        : num  70 35 61 57 49 53 57 44 70 29 ...
 $ sex_ps         : num  2 2 2 1 2 2 2 2 2 2 ...
 $ heart.disease  : num  1 0 0 0 0 0 0 0 0 1 ...
 $ renal.disease  : num  0 0 0 0 1 0 0 0 0 1 ...
 $ DM.uncontrolled: num  0 0 1 1 1 0 1 1 1 0 ...
 $ HT.uncontrolled: num  1 1 1 1 1 1 1 1 1 1 ...
 $ sistol         : num  140 150 140 200 160 160 150 140 200 150 ...
 $ diastol        : num  80 90 90 110 100 100 90 80 120 90 ...
 $ E              : num  3 4 4 4 4 4 4 3 4 4 ...
 $ M              : num  6 6 6 6 6 6 6 5 6 6 ...
```

```
 $ V.coherent     : num  1 1 1 1 1 1 1 0 1 1 ...
 $ as_urat        : num  4.8 5.2 5.8 5.2 4.6 5.9 5.5 3.7 5.3 9.4 ...
 $ imt            : num  26.2 30 21.8 28.8 23 26 26 29.3 24.9 38 ...
 $ lama_rawat     : num  5 4 3 4 6 4 5 4 4 3 ...
 $ nihss_msk      : num  10 0 2 5 4 3 12 3 10 3 ...
 $ dist           : num  18.46 8.84 6.54 7.02 15.14 ...
 - attr(*, "na.action")= 'omit' Named int [1:270] 4 11 23 28 29 35 62 63 64 71 ...
  ..- attr(*, "names")= chr [1:270] "4" "11" "23" "28" ...
```

The dependent variable was constructed as a `survival` object using the `outcome` and `lama_rawat` variables, each representing the patient outcome (discharged = 0, death = 1). The `survival` object is summarized in the following code chunk, where `+` indicates censoring.

```r
event <- with(clean_tbl, Surv(lama_rawat, outcome)) %T>%
  {print(table(.))}
```

```
.
  1  1+   2  2+   3  3+   4  4+   5  5+   6  6+   7  7+   8  8+   9  9+  10 10+
  1  21  20 188  14 672  14 946   9 526   8 331   4 161   6  80   7  57   1  31
 11 11+  12 12+  13 13+  14 14+  15 15+  16 16+  17 17+  18 18+  19 19+  20 20+
  1  39   1  21   1  11   5  13   3  11   1  11   3  10   4   6   2   3   2   3
 21 21+ 22+  23 23+ 24+  25 25+  26 26+  27 27+ 28+ 30+ 32+  33 39+ 49+
  1   4   2   2   1   3   1   1   1   2   1   1   4   2   1   1   1   1
```

The categorical variables, which will be used in the non-parametric model, is described as follow:

```r
cat_vars <- subset(clean_tbl, select = c(3:7, 10:12)) %>% names()

subset(clean_tbl, select = cat_vars) %>%
  lapply(table)
```

```
$sex_ps

   1    2 
1181 2097 

$heart.disease
```

```
   0    1
2361  917

$renal.disease

   0    1
2902  376

$DM.uncontrolled

   0    1
2194 1084

$HT.uncontrolled

   0    1
1022 2256

$E

   0    1    2    3    4    5
   1   13   44  181 3038    1

$M

   0    1    2    3    4    5    6
   1    6    4    5   33  259 2970

$V.coherent

   0    1
 459 2819
```

Kaplan-Meier Non-Parametric Statistics

We proceeded with a non-parametric approach using a Kaplan-Meier model, represented as a survival table spanning over 50-day of survival with a 10-day interval. The black line represents the first group of the categorical variables, e.g. black line in `sex_ps` represents the value `1` (female).

```
$`1`
   Characte    Time 0    Time 10    Time 20    Time 30    Time 40    Time 50
```

ristic						
Overall	100% (100%, 100%)	92% (89%, 94%)	64% (54%, 75%)	45% (33%, 63%)	30% (13%, 72%)	- (-, -)

Column names: label, stat_1, stat_2, stat_3, stat_4, stat_5, stat_6

$sex_ps

Characteristic	Time 0	Time 10	Time 20	Time 30	Time 40	Time 50
sex_ps						
1	100% (100%, 100%)	91% (87%, 96%)	63% (47%, 86%)	33% (15%, 76%)	- (-, -)	- (-, -)
2	100% (100%, 100%)	92% (89%, 95%)	64% (53%, 78%)	52% (38%, 71%)	52% (38%, 71%)	- (-, -)

Column names: label, stat_1, stat_2, stat_3, stat_4, stat_5, stat_6

$heart.disease

Characteristic	Time 0	Time 10	Time 20	Time 30	Time 40	Time 50
heart.disease						
0	100% (100%, 100%)	96% (94%, 98%)	76% (61%, 94%)	57% (36%, 90%)	38% (15%, 95%)	- (-, -)
1	100% (100%, 100%)	87% (83%, 91%)	57% (46%, 71%)	38% (24%, 61%)	- (-, -)	- (-, -)

Column names: label, stat_1, stat_2, stat_3, stat_4, stat_5, stat_6

$renal.disease

Characteristic	Time 0	Time 10	Time 20	Time 30	Time 40	Time 50
renal.disease						

```
   0          100%      92%      77%      64%      42%     - (-, -)
             (100%,    (90%,    (68%,    (47%,    (18%,
             100%)      95%)     88%)     86%)    100%)
   1          100%      87%      46%      25%     - (-, -)  - (-, -)
             (100%,    (82%,    (31%,    (12%,
             100%)      93%)     67%)     52%)

Column names: label, stat_1, stat_2, stat_3, stat_4, stat_5, stat_6

$DM.uncontrolled
   Characte    Time 0    Time 10   Time 20   Time 30   Time 40   Time 50
   ristic

   DM.uncon
   trolled
   0          100%      92%      69%      41%      27%     - (-, -)
             (100%,    (89%,    (57%,    (25%,    (11%,
             100%)      95%)     84%)     67%)     69%)
   1          100%      91%      57%      57%     - (-, -)  - (-, -)
             (100%,    (87%,    (42%,    (42%,
             100%)      95%)     76%)     76%)

Column names: label, stat_1, stat_2, stat_3, stat_4, stat_5, stat_6

$HT.uncontrolled
   Characte    Time 0    Time 10   Time 20   Time 30   Time 40   Time 50
   ristic

   HT.uncon
   trolled
   0          100%      90%      71%      54%     - (-, -)  - (-, -)
             (100%,    (86%,    (58%,    (35%,
             100%)      95%)     88%)     84%)
   1          100%      92%      61%      41%      41%     - (-, -)
             (100%,    (89%,    (49%,    (26%,    (26%,
             100%)      95%)     76%)     66%)     66%)

Column names: label, stat_1, stat_2, stat_3, stat_4, stat_5, stat_6

$E
   Characte    Time 0    Time 10   Time 20   Time 30   Time 40   Time 50
   ristic
```

E						
0	100% (100%, 100%)	- (-, -)	- (-, -)	- (-, -)	- (-, -)	- (-, -)
1	100% (100%, 100%)	65% (42%, 100%)	- (-, -)	- (-, -)	- (-, -)	- (-, -)
2	100% (100%, 100%)	77% (63%, 93%)	69% (52%, 92%)	69% (52%, 92%)	- (-, -)	- (-, -)
3	100% (100%, 100%)	79% (71%, 87%)	63% (47%, 86%)	51% (30%, 86%)	51% (30%, 86%)	- (-, -)
4	100% (100%, 100%)	94% (92%, 97%)	63% (51%, 79%)	41% (26%, 65%)	- (-, -)	- (-, -)
5	100% (100%, 100%)	- (-, -)	- (-, -)	- (-, -)	- (-, -)	- (-, -)

Column names: label, stat_1, stat_2, stat_3, stat_4, stat_5, stat_6

$M

Characteristic	Time 0	Time 10	Time 20	Time 30	Time 40	Time 50
M						
0	100% (100%, 100%)	- (-, -)	- (-, -)	- (-, -)	- (-, -)	- (-, -)
1	100% (100%, 100%)	75% (43%, 100%)	- (-, -)	- (-, -)	- (-, -)	- (-, -)
2	100% (100%, 100%)	38% (8.4%, 100%)	- (-, -)	- (-, -)	- (-, -)	- (-, -)
3	100% (100%, 100%)	80% (52%, 100%)	80% (52%, 100%)	- (-, -)	- (-, -)	- (-, -)
4	100% (100%, 100%)	63% (46%, 86%)	54% (35%, 83%)	54% (35%, 83%)	54% (35%, 83%)	- (-, -)
5	100%	85%	60%	36%	- (-, -)	- (-, -)

```
               (100%,     (79%,      (46%,      (19%,
               100%)       90%)       78%)       69%)
   6            100%       94%        61%        47%      - (-, -)   - (-, -)
               (100%,     (91%,      (47%,      (31%,
               100%)       97%)       80%)       70%)

Column names: label, stat_1, stat_2, stat_3, stat_4, stat_5, stat_6

$V.coherent
   Characte    Time 0    Time 10    Time 20    Time 30    Time 40    Time 50
   ristic

   V.cohere
   nt
   0            100%       85%        60%        38%        19%      - (-, -)
               (100%,     (81%,      (48%,      (22%,      (4.2%,
               100%)       90%)       73%)       66%)       84%)
   1            100%       94%        66%        50%      - (-, -)   - (-, -)
               (100%,     (91%,      (51%,      (33%,
               100%)       97%)       85%)       75%)

Column names: label, stat_1, stat_2, stat_3, stat_4, stat_5, stat_6


$`1`
  strata median lower upper
1    All     26    23    NA

$sex_ps
    strata median lower upper
1 sex_ps=1     26    19    NA
2 sex_ps=2     NA    21    NA

$heart.disease
           strata median lower upper
1 heart.disease=0     33    25    NA
2 heart.disease=1     23    20    NA

$renal.disease
           strata median lower upper
1 renal.disease=0     33    26    NA
2 renal.disease=1     20    18    NA
```

```
$DM.uncontrolled
             strata median lower upper
1 DM.uncontrolled=0     26    23    NA
2 DM.uncontrolled=1     NA    19    NA

$HT.uncontrolled
             strata median lower upper
1 HT.uncontrolled=0     33    23    NA
2 HT.uncontrolled=1     26    20    NA

$E
  strata median lower upper
1    E=0     NA    NA    NA
2    E=1     14     9    NA
3    E=2     NA    NA    NA
4    E=3     NA    20    NA
5    E=4     26    21    NA
6    E=5     NA    NA    NA

$M
  strata median lower upper
1    M=0     NA    NA    NA
2    M=1     15     5    NA
3    M=2      9     2    NA
4    M=3     NA    NA    NA
5    M=4     NA    10    NA
6    M=5     26    18    NA
7    M=6     23    20    NA

$V.coherent
        strata median lower upper
1 V.coherent=0     26    19    NA
2 V.coherent=1     NA    21    NA
```

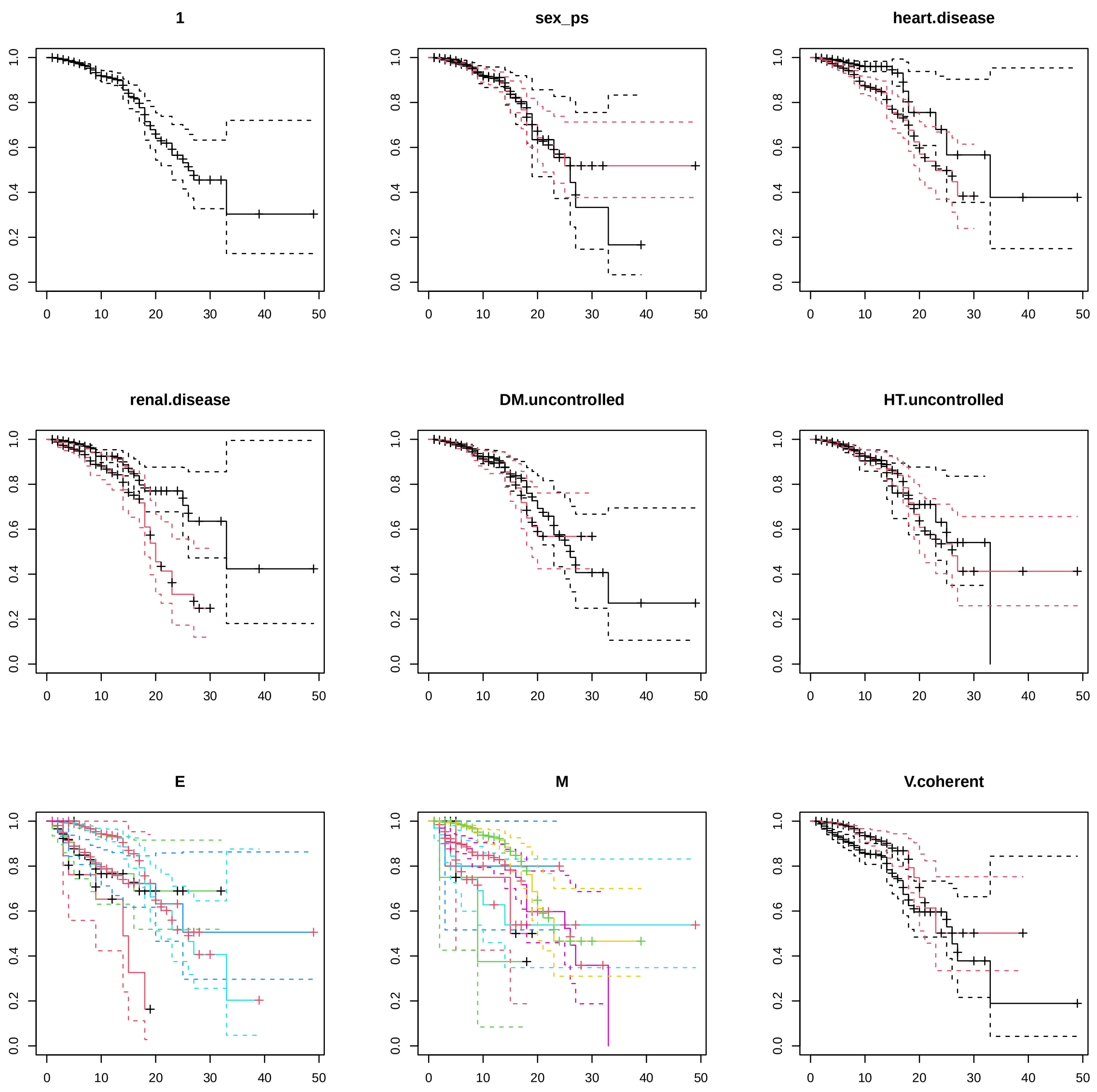

Figure 1: Survival plots

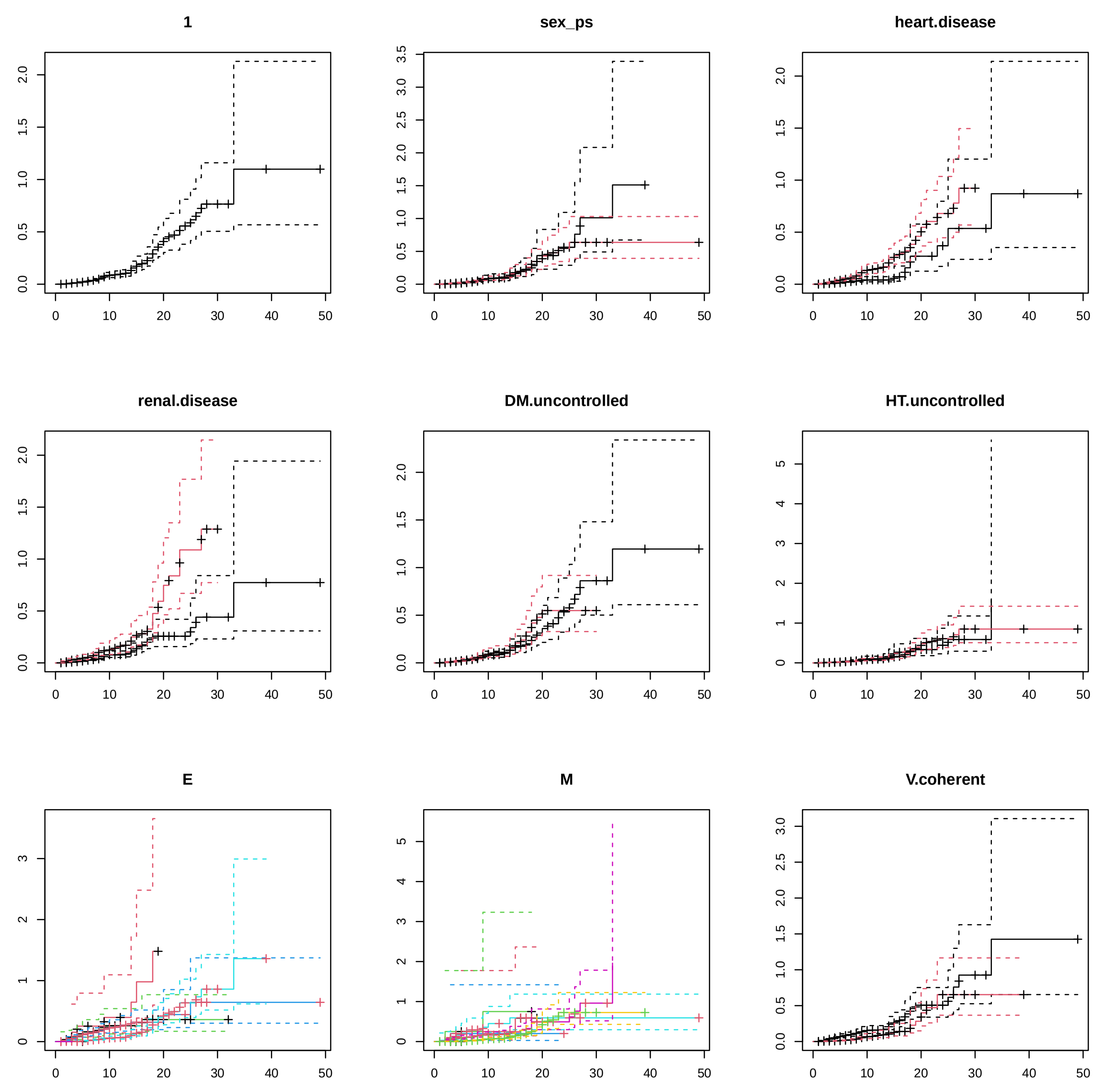

Figure 2: Cumulative hazard plots

Cox's Parametric Statistics

Previously, we fit several independent variables into a Cox's proportional hazard model. These variables were selected through a data mining process, where we fit in various logistic regression models and using Brier's score as a selection criteria. Variables we included in the final Cox's model were as follow:

```
winpred <- readRDS("../RData/winpred.Rds")

mod_cox <- subset(clean_tbl, select = c("lama_rawat", "outcome", winpred)) %>%
  {coxph(Surv(lama_rawat, outcome) ~ ., data = ., robust = TRUE, x = TRUE)}

mod_cox %>%
  gtsummary::tbl_regression(exponentiate = TRUE) %>%
  gtsummary::as_hux_table()
```

**Characteristic**	**HR**	**95% CI**	**p-value**
nihss_msk	1.06	1.03, 1.10	<0.001
heart.disease	1.90	1.23, 2.94	0.004
E	0.51	0.36, 0.73	<0.001
renal.disease	1.68	1.08, 2.62	0.021
imt	0.93	0.88, 0.99	0.013
umur_ps	1.03	1.01, 1.05	0.012
M	0.57	0.37, 0.88	0.011
sex_ps	1.43	0.96, 2.13	0.083
dist	0.98	0.96, 0.99	0.009
as_urat	1.11	1.05, 1.18	<0.001

HR = Hazard Ratio, CI = Confidence Interval

Furthermore, we evaluated the proportional hazard assumption. As a rule of thumb, variables with a proportional hazard will produce $p > 0.05$ and a seemingly linear hazard plot.

```
cox_assume <- cox.zph(mod_cox) %T>% print()
```

```
                 chisq df      p
nihss_msk      8.04456  1 0.0046
heart.disease  0.00545  1 0.9411
E              7.36992  1 0.0066
renal.disease  5.78604  1 0.0162
imt            0.10256  1 0.7488
umur_ps        3.53890  1 0.0599
M             11.24783  1 0.0008
sex_ps         1.96712  1 0.1608
dist           3.33536  1 0.0678
as_urat        1.99537  1 0.1578
GLOBAL        29.05495 10 0.0012
```

By evaluating the time-dependant β coefficient, we came to a conclusion that not all variables satisfy the proportional hazard assumption. Variables satisfying the assumption is denoted by p $>$ 0.05 and nearly linear in Figure 3 and Figure 4, such variables are including:

- `heart.disease`
- `imt`
- `umur_ps`
- `sex_ps`
- `dist`

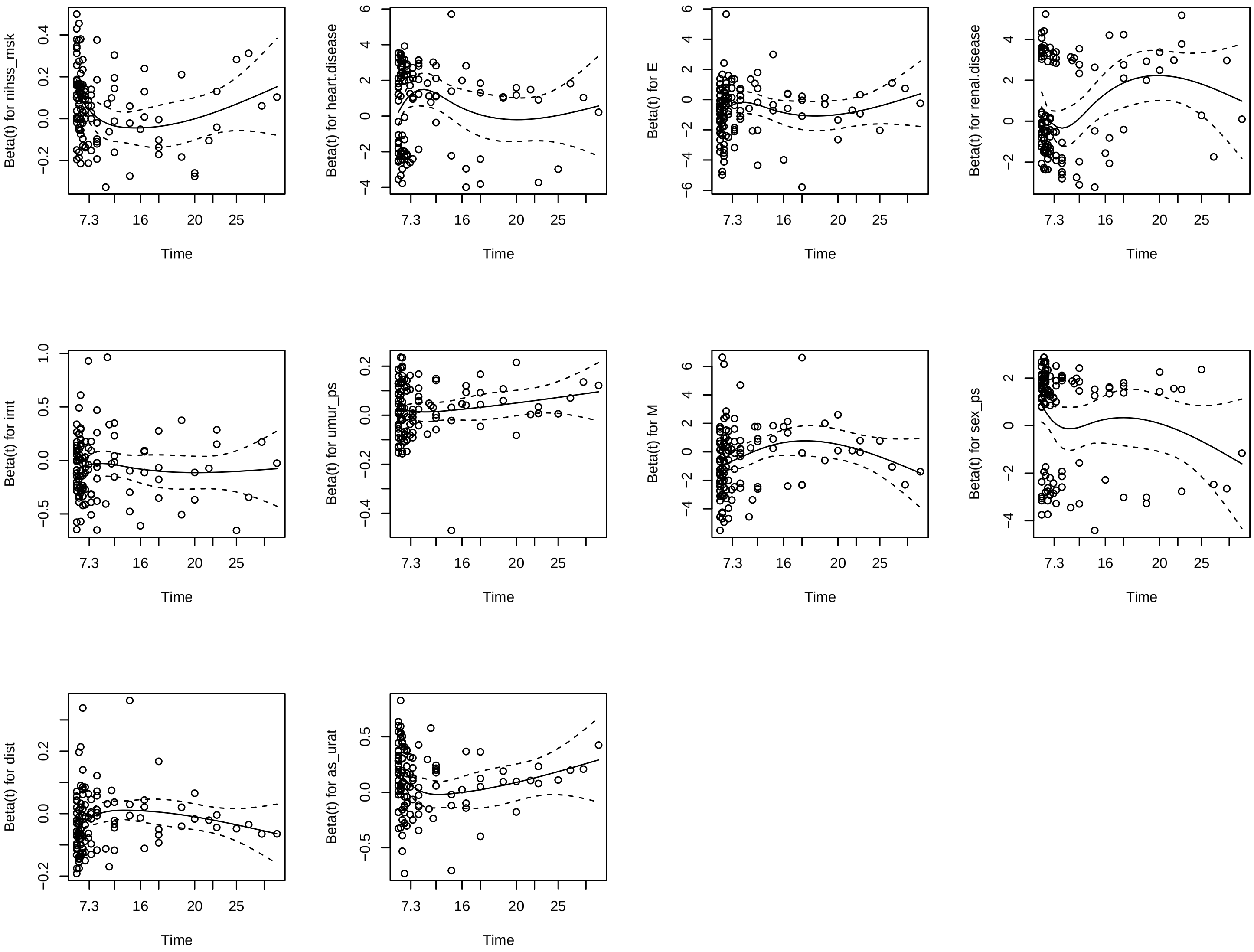

Figure 3: Proportional hazard assumption, should be seemingly linear

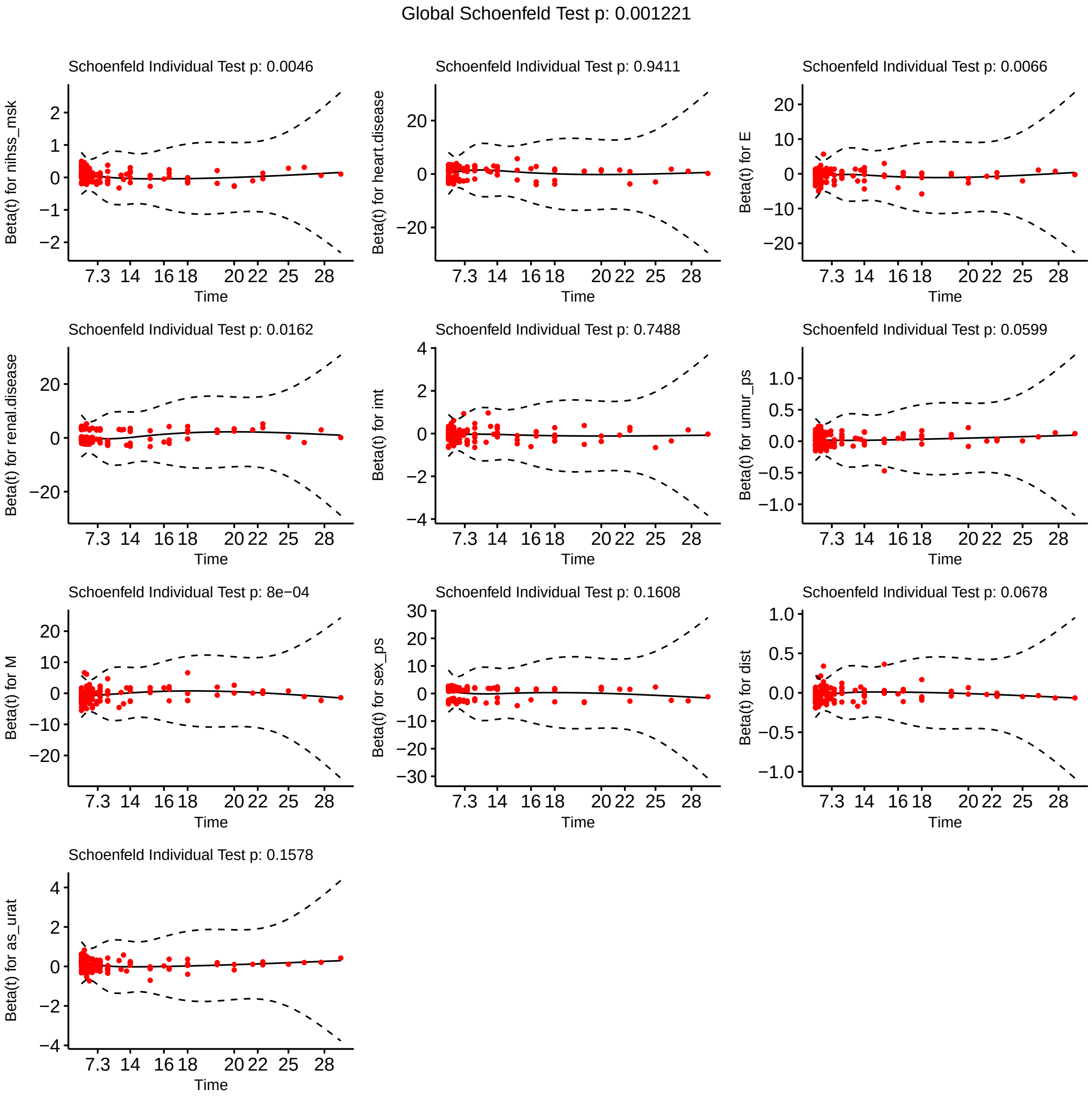

Figure 4: Proportional hazard assumption plotted with Schoenfeld residual

Refitting the Cox's Regression Model

Per previous evaluation, we refit the model using variables satisfying the proportional hazard assumption.

```r
mod_cox2 <- coxph(
  Surv(lama_rawat, outcome) ~ heart.disease + imt + umur_ps + sex_ps + dist,
  data = clean_tbl, robust = TRUE, x = TRUE
)

mod_cox2 %>%
  gtsummary::tbl_regression(exponentiate = TRUE) %>%
  gtsummary::as_hux_table()
```

**Characteristic**	**HR**	**95% CI**	**p-value**
heart.disease	2.56	1.69, 3.88	<0.001
imt	0.92	0.87, 0.97	0.002
umur_ps	1.02	1.00, 1.04	0.026
sex_ps	1.39	0.95, 2.04	0.090
dist	1.00	1.00, 1.00	<0.001

HR = Hazard Ratio, CI = Confidence Interval

```r
par(mfrow = c(2, 3))
cox.zph(mod_cox2)
```

```
               chisq df     p
heart.disease 0.9269  1 0.336
imt           0.1833  1 0.669
umur_ps       1.1784  1 0.278
sex_ps        3.9701  1 0.046
dist          0.0131  1 0.909
GLOBAL        5.0643  5 0.408
```

Since this model also yields non-proportional hazards, the final fit is as follow:

```r
mod_cox3 <- coxph(
  Surv(lama_rawat, outcome) ~ heart.disease + imt + umur_ps + dist,
  data = clean_tbl, robust = TRUE, x = TRUE
)

mod_cox3 %>%
  gtsummary::tbl_regression(exponentiate = TRUE) %>%
  gtsummary::as_hux_table()
```

**Characteristic**	**HR**	**95% CI**	**p-value**
heart.disease	2.56	1.68, 3.88	<0.001
imt	0.92	0.88, 0.97	0.002
umur_ps	1.02	1.00, 1.04	0.038
dist	1.00	1.00, 1.00	<0.001

HR = Hazard Ratio, CI = Confidence Interval

```r
par(mfrow = c(2, 2))
cox.zph(mod_cox3)
```

```
               chisq df    p
heart.disease 0.8358  1 0.36
imt           0.1962  1 0.66
umur_ps       1.0038  1 0.32
dist          0.0263  1 0.87
GLOBAL        2.3317  4 0.67
```

Exploring the Competing Risk

This dataset does not contain any other outcome status other than `0` (discharged) and `1` (death). However, when considering the presence of competing risk, technically we can evaluate the cumulative incidence as shown in Figure 5.

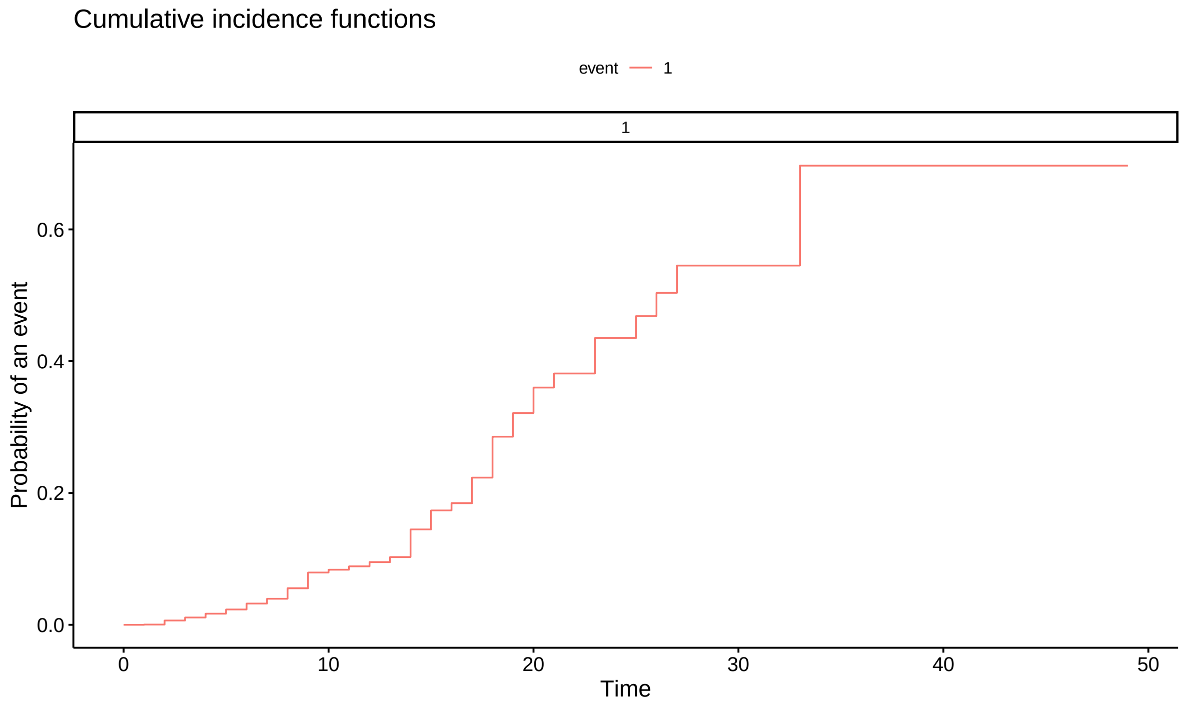

Figure 5: Cumulative incidence function of post-stroke mortality

When mining the data, we have several grouping variables which could be of interest when determining death, including:

- `sex_ps`
- `heart.disease`
- `renal.disease`
- `DM.uncontrolled`
- `HT.uncontrolled`

Incorporating these groups, we can evaluate the cumulative incidence function as shown in Figure 6.

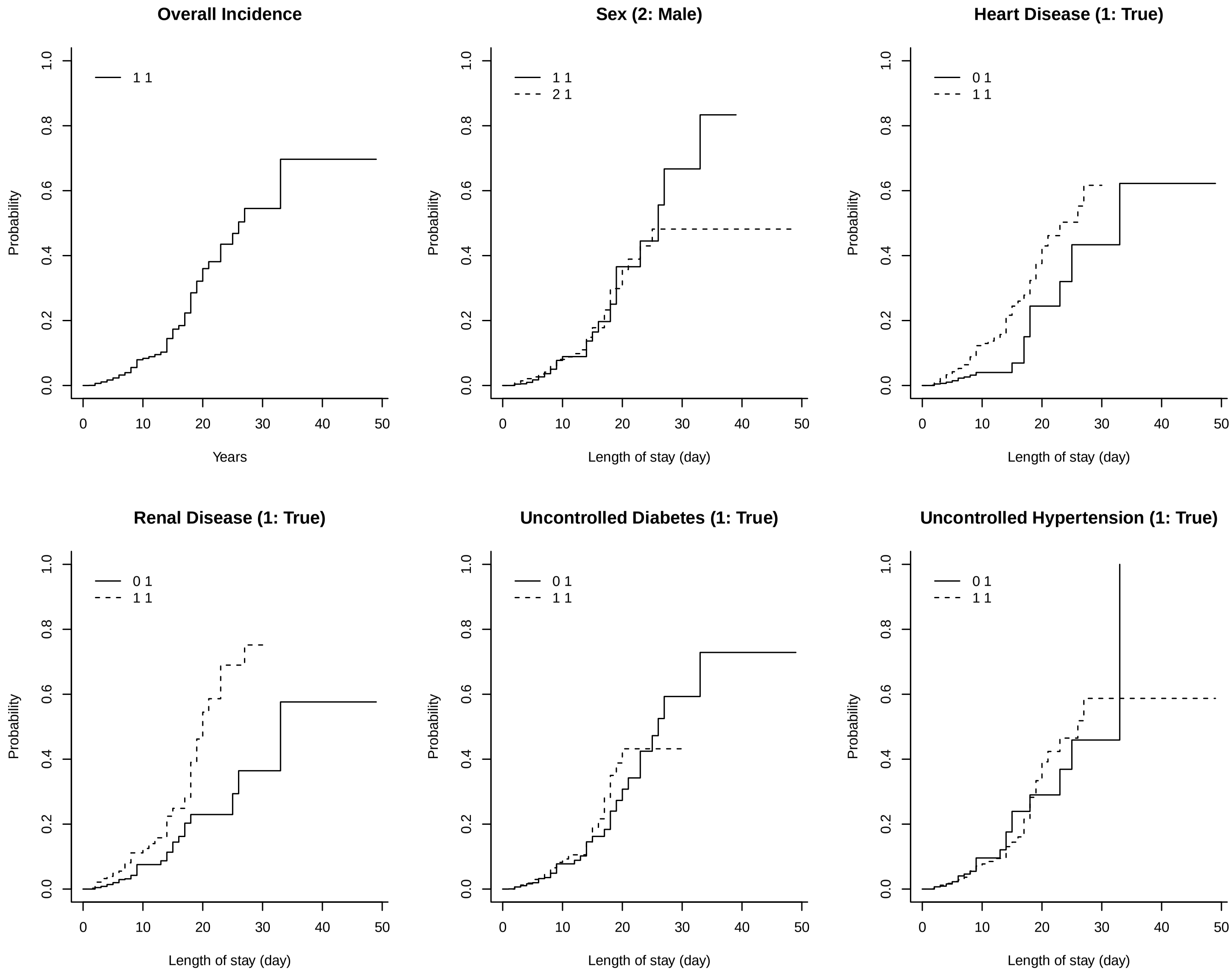

Figure 6: Cumulative incidence function over different groups

Fine-Gray Subdistribution Hazard Model

```r
mod_shr <- subset(clean_tbl, select = c("lama_rawat", "outcome", winpred)) %>%
  {crr(Surv(lama_rawat, as.factor(outcome)) ~ ., data = .)}
```

Table 2: Regression on the full model

(a) Fine-Gray Model

Characteristic	HR	95% CI	p-value
nihss_msk	1.06	1.03, 1.10	<0.001
heart.disease	1.90	1.23, 2.92	0.004
E	0.52	0.36, 0.74	<0.001
renal.disease	1.66	1.08, 2.57	0.022
imt	0.93	0.88, 0.98	0.011
umur_ps	1.03	1.01, 1.05	0.012
M	0.58	0.38, 0.88	0.010
sex_ps	1.43	0.96, 2.12	0.079
dist	0.98	0.96, 0.99	0.008
as_urat	1.11	1.05, 1.18	<0.001

(b) Cox's PH regression

Characteristic	HR	95% CI	p-value
nihss_msk	1.06	1.03, 1.10	<0.001
heart.disease	1.90	1.23, 2.94	0.004
E	0.51	0.36, 0.73	<0.001
renal.disease	1.68	1.08, 2.62	0.021
imt	0.93	0.88, 0.99	0.013
umur_ps	1.03	1.01, 1.05	0.012
M	0.57	0.37, 0.88	0.011
sex_ps	1.43	0.96, 2.13	0.083
dist	0.98	0.96, 0.99	0.009
as_urat	1.11	1.05, 1.18	<0.001

Fine-Gray Subdistribution Hazard Model with Refitted Variables

```r
mod_shr2 <- crr(
  Surv(lama_rawat, as.factor(outcome)) ~ heart.disease + imt + umur_ps + sex_ps + dist,
  data = clean_tbl
)
```

Table 3: Regression with refitted variables

(a) Fine-Gray Model

Characteristic	HR	95% CI	p-value
heart.disease	2.55	1.69, 3.86	<0.001
imt	0.92	0.88, 0.97	0.002
umur_ps	1.02	1.00, 1.04	0.026
sex_ps	1.39	0.95, 2.03	0.089
dist	1.00	1.00, 1.00	<0.001

(b) Cox's PH regression

Characteristic	HR	95% CI	p-value
heart.disease	2.56	1.69, 3.88	<0.001
imt	0.92	0.87, 0.97	0.002
umur_ps	1.02	1.00, 1.04	0.026
sex_ps	1.39	0.95, 2.04	0.090
dist	1.00	1.00, 1.00	<0.001

```r
mod_shr3 <- crr(
  Surv(lama_rawat, as.factor(outcome)) ~ heart.disease + imt + umur_ps + dist,
  data = clean_tbl
)
```

Table 4: Regression with refitted variables, removing `sex_ps`

(a) Fine-Gray Model

**Characteristic**	**HR**	**95% CI**	**p-value**
heart.disease	2.55	1.68, 3.86	<0.001
imt	0.92	0.88, 0.97	0.002
umur_ps	1.02	1.00, 1.04	0.038
dist	1.00	1.00, 1.00	<0.001

(b) Cox's PH regression

**Characteristic**	**HR**	**95% CI**	**p-value**
heart.disease	2.56	1.69, 3.88	<0.001
imt	0.92	0.87, 0.97	0.002
umur_ps	1.02	1.00, 1.04	0.026
sex_ps	1.39	0.95, 2.04	0.090
dist	1.00	1.00, 1.00	<0.001
