# Supplementary material for: Prognostic model of in-hospital ischemic stroke mortality based on an electronic health record cohort in Indonesia
Source: PLoS One. 2024 Jun 12;19(6):e0305100. doi: 10.1371/journal.pone.0305100 (PMC11168658; doi:10.1371/journal.pone.0305100)
Supplement: Attachment — Submitted filename: Response to Reviewers Rebuttle Paper 1 B-2_final.docx [file pone.0305100.s002.docx]

**Response to Reviewers**

**Reviewer #1: thank you for the review chance. I would like to recommend the inclusion of thrombophilia types [e.g. protein C, protein S, antithrombin deficiency ..etc] as well as inclusion of other prognostic scales " e.g. THRIVE and Orpington scales...etc" as the Asian population has higher incidence of small-vessel ischemic stroke and stroke occurrence in younger ages as compared to Caucasian population, partly due to thrombophilia etiologies.**

We thank the reviewer for this important comment and agree that the suggested prognostic scales and thrombophilia types are important for Asian population. Sadly, we do not have the necessary information. Hence, we did not include nor mention any of those data in our manuscript.

**Reviewer #2: In this manuscript entitled 'Prognostic Model of In-hospital Ischemic Stroke Mortality Based on an Electronic Health Record Cohort in Indonesia' the authors investigate the predictors of in-hospital mortality in patients with ischemic stroke (IS). The study is based on the IS medical records from the National Brain Centre Hospital, Jakarta, Indonesia with a total of 3278 patients with IS. The authors worked on an interesting topic regarding public health meassage and local regional South-east Asia countries but, in my opinion, there are some methodological issues and suggestions to consider.**

**- there is a discrepancy between the period in which the study was conducted (one would imagine operationally) and the period of observation and date collection, respectively October 2021 to February 2022 versus years 2019-2020 (without specifying exact beginning and end). The authors should better explain this difference and possibly consider the possibility, if any, of extending the observation period. It would also be useful for the date collection to have a precise observation period (not only year but also month and/or day).**

We acknowledge that the statement of operational study duration and data collection duration led to confusion. To avoid this problem, we removed the line about operational study duration in paragraph 1 of methods section. We apologize for the wrong data collection period written in paragraph 4 of methods section, we changed the data collection years in the line and added the specific start and end date of data collection duration.

Old line: “*Data for a period of one year were collected from 2019 to 2020.*”

New line: “*Data for a period of one year was collected from 1 January to 31 December 2020.*”

**- As far as the outcome under analysis is concerned, it is presumable that the cases were identified using the ICD definition. This should be made explicit.**

We realize that we did not write it clearly that the diagnosed cases were recorded in medical record along with their ICD definition. We revised the following line in the first paragraph of methods section:

Old line: “*The study population included hospitalised IS patients according to the Trial of Org 10172 in Acute Stroke Treatment (TOAST) definition.*”

New line: “*The study population included hospitalised IS patients according to the Trial of Org 10172 in Acute Stroke Treatment (TOAST) definition along with the ICD-10 code I63 for acute ischemic stroke.*”

**- It is unclear whether the mortality assessment was only carried out intra-hospital. Did the observation continue for some time outside the hospital as well?**

Our apologies for not stated it in the methods section that we only assessed mortality during hospitalization. We added the necessary information in the first line of **Clinical outcomes** of methods section.

Old line: “The clinical outcomes were all-cause and stroke-related mortality rates.”

New line: “The clinical outcomes were all-cause and stroke-related mortality rates during hospitalization.”

**- The time-to-event analysis should be enriched with a competitive risk analysis as discharge represents an event that can drastically change the patients' medical history. Therefore not only simple COX but also cumulative incidence function curves and the Fine and Gray model (Resche-Rigon M et al. Crit Care. 2006 Feb;10(1):R5).**

We thank the reviewer for raising the issue about competing risk. We added the cumulative incidence function curves and the comparison of Cox regression results against Fine and Gray models in the results section.

**- The authors claim to have removed all records with missing data. Before simple removal it would be appropriate to investigate the significance of this missingness using appropriate analyses (pattern analysis, missing per patient, missing per item, missing by outcome). It is evident that, for example, a reduction in the number of subjects leads to a change in the incidence of outcomes (from 4.69% mortality to 3.48%), so it is assumed that in the 7.9% of missing data there is a different percentage of subjects with an unfavourable outcome. Could this have introduced a bias into the analysis?**

Indeed, we took into account the possibility of bias being introduced in this analysis. We thoroughly explored the missingness to assess the mechanism, i.e. MCAR/MAR/MNAR. It’s understandably challenging to differentiate MAR/MNAR through statistical analysis procedure; and it is more practical to determine whether the missing values occurred due to MCAR/non-MCAR, which could be MAR/MNAR. For clinical research, complete-case analysis in datasets with MAR entries still produces valid results (read more: <https://doi.org/10.1016/j.jclinepi.2022.08.016>). In another review of published RCTs, the majority of published studies still used complete case-analysis by discarding entries with null values (read more: <https://doi.org/10.1186/1471-2288-14-118>). Meanwhile, another review also suggested excluding observation in case of missingness in primary predictors for studies with cross-sectional design (read more: <https://doi.org/10.1016/j.pmrj.2015.07.011>). As such, we concluded that using a complete-case analysis is still arguably sound and justified. We attached our exploration on missingness in our supplementary materials, page 2-12.

**- The authors state that they selected the variables using a stepwise regression, forward or backward? Furthermore, they mention the use of other classification models compared with appropriate metrics. Both models and metrics should be listed and in a hypothetical supplementary material, the results of these comparisons should be presented to see if the type of algorithm may have influenced the final selection of variables.**

We agree that more detail about the analysis should be added as supplementary. We attached the supplementary about the detail of our analysis along with our revised manuscript.

We used a bidirectional stepwise regression, a combination of forward and backward selection. We added this information in the first paragraph of **Statistical analysis**.

Old line: “To aid the exploratory procedure, significant variables reported in previous studies are included in stepwise regression models.”

New line: “To aid the exploratory procedure, significant variables reported in previous studies are included in stepwise regression models using a combination of forward and backward selection.”

**- Table 1 should also present the overall data and not only the comparison between subjects with and without an event.**

We added a column in Table 1 that showed the baseline characteristic for the overall patients in our manuscript.

**- Was the GCS used as a continuous variable or category? Shouldn't it be a continuous score?**

We understand that the GCS should be a continuous variable however the clinicians only assessed eye and movement scores of GCS as continuous measurement while the verbal as either coherence or incoherence. Thus, we decided to include GCS in the analysis separately for eye, movement, and verbal assessment.

**- The figure presented represents the survival plot as estimated from the Cox's regression model. The time-to-event variable should be described a priori without any modelling. The overall kaplan meier, median follow-up, median survival and confidence interval should be presented. This would help readers to better understand the type of patients and the time frame studied.**

Thank you for the suggestion, we have added the descriptive statistics in our supplementary file from page 22 to 29.
