# Supplementary material for: Prognostic model of in-hospital ischemic stroke mortality based on an electronic health record cohort in Indonesia
Source: PLoS One. 2024 Jun 12;19(6):e0305100. doi: 10.1371/journal.pone.0305100 (PMC11168658; doi:10.1371/journal.pone.0305100)
Supplement: Attachment — Submitted filename: Response to Reviewers Rebuttal Paper 1 B 2023-11-27.docx [file pone.0305100.s003.docx]

**Response to Reviewers**

**Reviewer #3: The authors present a retrospective observational study used IS medical records from a single (tertiary, high volume) center in Indonesia, with predictors of in-hospital mortality in patients with IS.
I would suggest the authors to consider the following suggestions:
- having the manuscript checked by a native English speaker for errors regarding grammar, synthax etc.
- having a more focused and structured discussion section.**

We thanked reviewer for the comment. We revised discussion section to make the structure better and more focused. Further, we asked professional proofreader to proofread our manuscript after we addressed comments from all reviewers.

**Reviewer #4:  Major Criticism
Do the authors explore in deep all cause of mortality? Indeed, Ischemic Stroke has several complications, including infections?
indeed mortality after ischemic stroke other than metabolic or cardiac causes has other factors as infection well known since a decade: "Post-stroke infection: A systematic review and meta-analysis 10.1186/1471-2377-11-110" particularly pneumonia "
Int J Stroke. 2019 Feb;14(2):125-136. doi: 10.1177/1747493018806196"
Therefore please specify if you can have access or not to ICD classification on Clinical dismission records

Further it would be useful also estimate how many patients had previous hospital access.

Minor Criticism
improve material and methods better clarifying the data extraction method and model on ICD evaluation, for instance you should report what kind of code you explored and why you selected those.
Improve tables in terms of better details about previous hospital access and cause of mortality.**

We appreciated the reviewer’s inputs to improve our manuscripts. We went back to our medical record to get the related data, however due to time constraint we could only get aggregate data. It was also very hard for us to get specific ICD 10 for cause of death because clinical autopsy is not compulsory procedure in Indonesia.

499 (14%) patients were referred from other hospital and the prominent cause of death was cardiac arrest, followed by pneumonia, and sepsis. We decided to add these data in the first paragraph of results section, not in table 1 because we only have aggregate data.

The following lines were added the first paragraph of results section:

*Around 14% of the patients (499 out of 3,561) were referred from other previous hospitals. The top three causes of death were cardiac arrest, pneumonia, and sepsis*.
