# Supplementary material for: Prognostic model of in-hospital ischemic stroke mortality based on an electronic health record cohort in Indonesia
Source: PLoS One. 2024 Jun 12;19(6):e0305100. doi: 10.1371/journal.pone.0305100 (PMC11168658; doi:10.1371/journal.pone.0305100)
Supplement: Attachment — Submitted filename: Response to Reviewer.docx [file pone.0305100.s004.docx]

**Response to reviewers:**

1. **Subject Selection Process
   The process of selecting subjects should be presented more clearly than the variable extraction process to reduce selection bias. Exclusion criteria should be defined and the reasons for exclusion should be explained. It should also be clearly indicated how many subjects were excluded in the process. Based on the information provided in the text alone, it is difficult to determine which group was ultimately included in the analysis. Especially, since there were quite a few subjects without blood glucose data, it is unclear whether they were all excluded from the analysis.

   If the target group is not transparently defined, the results are difficult to trust as they may reflect analysis of a heterogeneous population.**

We thank the reviewer for highlighting the problem in understanding the patient’s selection process for our analysis. We replaced figure 1 with STROBE flowchart to make it easier to understand how we came to the final number of 3,278 patients for the analysis.

2. **Adjustment for Comorbidities
   Adjustment for comorbidities is necessary using a comorbidity index. If obtaining variables for the comorbidity index is difficult, it is necessary to investigate and mention the major comorbidities. Adjustment should be made based on the number of comorbidities, or alternatively, adjustment should be made for major comorbidities.

   Especially, since the data was collected during the Covid-19 pandemic, Covid-19-related deaths could have had a significant impact, so this should be taken into consideration.**

We agree that comorbidities are important risk factors. We added pneumonia and sepsis into the analysis and revised the tables, results, and discussion to accommodate the new results.

However, we could not add COVID-19 diagnosis as one of comorbidities into the analysis because only 13 patients were diagnosed with COVID-19 and none of them died. We added this into the limitation of our study.

3. **Co-variants Definition
   There is no accurate definition for heart disease, renal disease, uncontrolled diabetes, uncontrolled hypertension, etc.**

We apologize that we did not write a clear definition for uncontrolled diabetes and uncontrolled hypertension. We added the definition for those 2 terms under the paragraphs (highlighted in yellow) for the definition of heart disease and renal disease. Additionally, diagnosis of cardiovascular disease and renal disease were determined using the International Classification of Diseases, 10^th^ revision (ICD-10) as a standard operational procedure in our hospital, as presented in Methods section.

4. **Sex as Predictors for Mortality
   If sex becomes an important factor, additional analysis should be conducted by dividing by gender to determine if there are differences in factors related to post-stroke mortality according to sex.**

Thanks indeed for your suggestion, and in addressing your valuable suggestion we already performed the Cox-regression model and Fine-gray model as well, to adjust the confounders. These were presented in Table-2 A and table-2B.

5. **Figure 2 Needs Modification
   It seems that Figure 2 needs to be revised. Is the x-axis in Figure 2(A) correct? Is it "year" or "length of stay"? Although it is not an observation for 50 years, the graph indicates that it has been tracked for 50 years.

   The intended message in Figure 2(B) is not well understood. It's not clear what is being explained in the survival analysis. It would be helpful if the rationale for showing the survival curve in the manuscript is well explained.**

Once again, we thank the reviewer for pointing out the mistake in Figure 2A. The correct x-axis should be “length of stay”, not “year”. We replaced the figure with the correct one.

We agree with the reviewer that the title for Figure 2B was not clear enough.

We changed the title into:
Figure 2B Estimated survival probability based on Cox’s regression model result in table 2B.

6. **Other Comments
   1) The lack of explanations for tables and figures in the text makes it difficult to match them with the content and lowers comprehensibility.
   2) It is necessary to mention the possibility of more intensive drug therapy for patients with severe conditions who had low blood triglyceride levels, low-density lipoprotein (LDL) levels, and total cholesterol.
   3) It should be noted that the analyzed data represent only one year and may not reflect long-term trends, and that the data collected from the hospital may be limited in terms of external environmental factors or additional factors affecting patients.**

Thanks indeed for your valuable suggestions

- We already added the explanation within the text (Results section#) to improve comprehensibility as suggested.
- The treatment strategy was applied in out hospital based on the clinical practice guidelines, particularly for treating dyslipidemia.
- We added into limitation that our data could not be generalized to other populations for multiple reasons, including that it only represented 1 year period.
