# Supplementary material for: Prognostic model of in-hospital ischemic stroke mortality based on an electronic health record cohort in Indonesia
Source: PLoS One. 2024 Jun 12;19(6):e0305100. doi: 10.1371/journal.pone.0305100 (PMC11168658; doi:10.1371/journal.pone.0305100)
Supplement: Attachment — Submitted filename: Response to Reviewers.docx [file pone.0305100.s005.docx]

Dear Reviewers,

Thank you for your valuable feedback on our manuscript. We have carefully considered your comments and made the necessary revisions to improve the clarity and accuracy of our paper.

Specifically, you mentioned the need for labeling the X-axis in Figure 2-B. We have addressed this concern and have now labeled the X-axis as "Length of Day." We also have revised label of predictors in Figure-2A. This label accurately reflects the data presented in the figure and ensures that the information is clear and interpretable for our readers.

Additionally, we have incorporated some useful information and enhanced the clarity of the manuscript based on your suggestions.

We appreciate your attention to detail and believe that these revisions enhance the overall quality of our manuscript. Please find the revised figure included in the updated manuscript.

Thank you once again for your insightful suggestions.

Sincerely,
